# Association between triglyceride glucose index and severity of diabetic foot ulcers in type 2 diabetes mellitus

**DOI:** 10.1186/s13047-023-00663-7

**Published:** 2023-10-05

**Authors:** Weihao Chen, Xuedong Wang, Qilin Jiang, Jiyan Wu, Wanyan Shi, Xiaoxiao Wang, Yihu Yin, Jiayin Zheng, Xiang Hu, Cai Lin, Xingxing Zhang

**Affiliations:** 1 Department of Burn and Wound Center, The First Affiliated Hospital of Wenzhou Medical University, Shangcai Village, Wenzhou, 325000 Zhejiang China; 2 Department of Endocrinology and Metabolism, The First Affiliated Hospital of Wenzhou Medical University, Shangcai Village, Wenzhou, 325000 Zhejiang China; 3 Department of Internal Medicine, Taishun County Hospital of Traditional Chinese Medicine, Wenzhou, Zhejiang China; 4 Department of Endocrine, Wencheng People’s Hospital, Wenzhou, Zhejiang China; 5 Department of Clinical Laboratory, The First Affiliated Hospital of Wenzhou Medical University, Wenzhou, Zhejiang China

**Keywords:** Diabetic foot ulcer, Triglyceride glucose index, Chronic wounds

## Abstract

**Background:**

Triglyceride glucose (TyG) index is a good surrogate biomarker to evaluate insulin resistance (IR). The study aimed to investigate whether the TyG index is related to the severity of diabetic foot ulcers (DFUs) in patients with type 2 diabetes mellitus (T2DM).

**Methods:**

A total of 1059 T2DM patients were enrolled in this observational, retrospective, single-center study. TyG index was calculated as ln[fasting triglycerides (mg/dl) × fasting glucose (mg/ dl)/2]. The severity of DFUs was classified into mild-to-moderate DFUs (Wagner grade score < 3) and severe DFUs (Wagner grade score ≥ 3) based on Wagner classification. Patients were stratified according to the tertiles of TyG index. Logistic regression models were implemented to explore the association between TyG index and the severity of DFUs. Subgroup analyses were used to verify the reliability of results.

**Results:**

Compared with the reference lowest TyG tertile (T1), the highest tertile (T3) was associated with 0.377-fold increased risk of prevalence of severe DFUs (odds ratio [OR] 1.377, 95% confidence interval [CI] 1.017–1.865) (*P* = 0.039). After adjusting for potential confounders, the multivariable-adjusted OR and 95% CI were 1.506 (1.079–2.103) (*P* = 0.016) in patients with highest tertile. Moreover, subgroup analyses indicated that the association was stronger among men, patients with age ≥ 65 years, duration of diabetes more than 10 years, or without PAD.

**Conclusions:**

Elevated TyG index is independently associated with severity of DFUs even after adjusting conventional confounders.

## Introduction

Diabetic foot ulcer (DFU), a severe complication with diabetes, usually occurs due to neuropathy and peripheral arterial disease (PAD) with a high global prevalence of 6.3% [1, 2]. Nearly 15% patients with diabetes were suffering a foot ulcer and the amputation rate of patients with diabetes is 15- to 40-fold higher compared to general population, contributing to a high rate of non-traumatic lower extremity amputations [3, 4]. Identifying patients at risk of lower extremity amputation and early intervention are a concern for clinicians.

Insulin resistance (IR) means that insulin exerts unsatisfactory biological effect in both experimental and clinical conditions. Previous studies have demonstrated that IR is one of the essential risk factors for cardiovascular disease [5], such as hypertension, vascular stiffness, as well as related to the progression of atherosclerosis [6, 7]. And it also can be closely associated with an advancing chronic kidney disease progress. [8, 9]. Hyperglycemia originated from IR in patients with diabetes are predisposed to developing chronic wounds such as DFUs. Because IR could delay wound healing though interplay among impaired tissue regeneration, vasculopathy, neuropathy and inflammation [10, 11].

Triglyceride glucose (TyG) index recently is used as a surrogate biomarker to evaluate IR due to it availability and inexpensiveness [12]. Hyperinsulinemic-euglycemic clamp (HIEC), as the gold standard to identify IR, however, was not put into practice as often because of its time-consuming and expensive [13]. And studies have shown that TyG index has a good correlation with IR assessed by HIEC and homeostasis model assessment of insulin resistance (HOMA-IR) [14, 15]. A reaseach conducted by Vasques, A.C., et showed that TyG index had a better perfomance in predicting insulin resistance than HOMA-IR in a Brazilian population. [16]

To the best of our knowledge, the correlation between TyG index and severity of DFU is still unclear. Therefore, in this study we aim to clarify the relationship between the severity of DFU and TyG index in Southeast of China.

## Method

### Study population

**Fig. 1:**
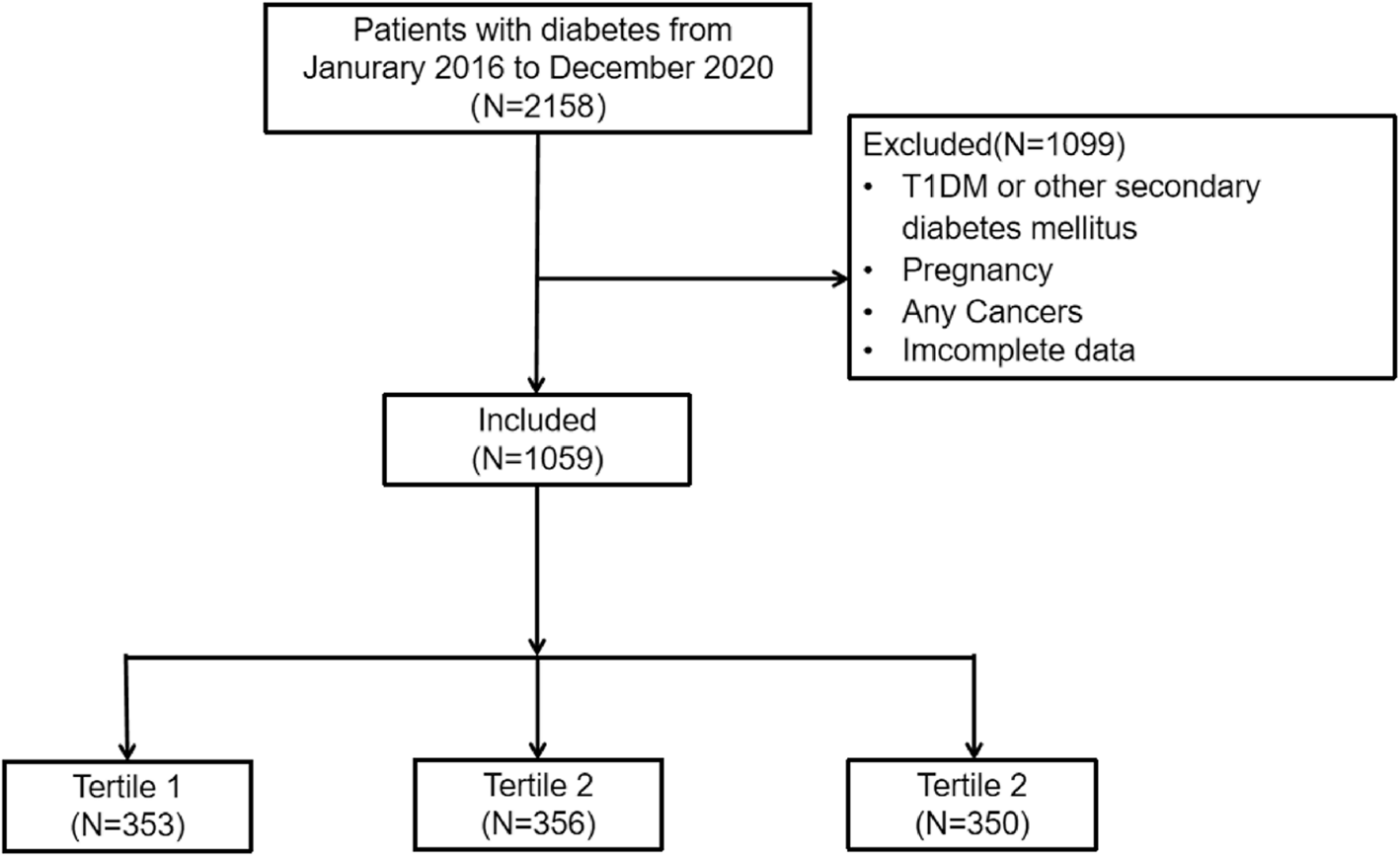
Flow chart of the study. *T1DM,* type 1 diabetes mellitus

Two thousand one hundred fifty-eight patients with diabetic foot ulcer (DFU) were retrospectively enrolled at the First Affiliated Hospital of Wenzhou Medical University between January 2016 to December 2020. A total of 1100 patients were excluded due to following reasons: (a) patients were diagnosed with other types of diabetes mellitus, (b) patients were combined with other short-term life-threatening conditions, such as cancer, (c) medical records were incomplete or inaccurate (Fig. 1).

### Data collection and definitions

Data on eligible patients were retrospectively extracted from the electronic medical system. Characteristics at admission, including age, gender, smoking history, drinking history, and body mass index (BMI). Peripheral venous blood samples including hemoglobin (Hb), albumin (ALB), HbA1c, total cholesterol (TC), triglyceride (TG), high-density lipoprotein cholesterol (HDL-C), low-density lipoprotein cholesterol (LDL-C) were collected after overnight fasting (> 8 h) and measured in hospital laboratory department. Equation of modification of diet in renal disease (MDRD) [17] was used to calculate estimated glomerular filtration rate (eGFR). Bilateral lower extremity arteries including femoral artery, superficial femoral artery, popliteal artery, anterior tibial artery, posterior tibial artery, and dorsalis pedis artery of patients were examined by duplex ultrasonography and patients who had intima-media thickness, plaque occurrence, stenosis, and filling defects in blood flow was defined as PAD and diagnosed by qualified, experienced ultrasonic diagnostic experts [18]. The degree of severity of DFUs was subdivided according to the Wagner classification (grade score 0–5) and categorized as mild-to-moderate DFUs (Wagner grade score < 3) and severe DFUs (Wagner grade score ≥ 3) [19]. The TyG index was calculated as ln[fasting triglycerides (mg/dl) × fasting glucose (mg/ dl)/2].

### Data analysis

Data normality was tested by Kolmogorov–Smirnov test. Continuous variables, described as mean ± standard deviation or median (interquartile range), were compared using by one-way analysis of variance (ANOVA) or Kruskal Wallis. And categorical variables, expressed as number (percentage), were compared using Chi-squared test or Mantel–Haenszel χ2 test. To estimate the association between TyG quartiles and Wagner classification(Wagner < 3 versus Wagner ≥ 3), logistic regression models were employed calculate odds ratios (ORs) and 95% confidence interval (CI). Finally, four multivariable regression models were constructed. Model 1 was not adjusted. Model 2 was adjusted for age, and gender. Model 3 was adjusted for variables included in Model 2 and BMI, smoking, alcohol, PAD, duration of diabetes ≥ 10 years. Model 4 was adjusted for variables included in Model 3 and Hb, ALB, HbA1c, eGFR, TC, LDL-C, HDL-C. Subgroup analyses were implemented to further verify the robustness of the results. What’s more, the interaction between subgroups was conducted to assess the interaction among subgroups. All statistical analyses were performed using SPSS for Windows version 22, and a two-sided *P* < 0.05 was considered as significant.

## Results

### Baseline characteristics

The clinical characteristics of the study population were stratified into three groups according to their TyG index levels (tertile 1: ≤ 7.28, tertile 2: 7.28 ~ 7.90, tertile 3: > 7.90). The baseline characteristics of the study population were shown in Table 1. Significant difference was observed in patients with PAD (*P* = 0.004). Among the 1059 patients, 756 patients had PAD as described above while the rest was normal in the lower limb arteries. Further Significant difference was also observed high score DFUs (*P* = 0.039). The highest tertile tended to have higher Alb (*P* < 0.001) and lower eGFR (*P* < 0.001). Furthermore, higher levels of HbA1c, TC, LDL-C were seen in the highest tertile.

**Table 1:** Clinical characteristics according to the TyG tertiles

Variables	Tertile 1(*N* = 353)	Tertile 2(*N* = 356)	Tertile 3(*N* = 350)	*p* value
Age (years)	68 (59,74)	68 (59,75)	66 (58,73)	0.298
Male, n (%)	231 (65.4)	238 (66.9)	211 (60.3)	0.16
BMI (kg/m2)	23.7 (21.6,25.1)	23.4 (21.5,25.9)	23.4 (21.6,25.7)	0.917
Smoking, n (%)	95 (26.9)	118 (33.1)	90 (25.7)	0.418
Drinking, n (%)	94 (26.6)	116 (32.6)	81 (23.1)	0.067
PAD, n (%)	263 (74.5)	266 (74.7)	227 (64.9)	0.004
DFUs				0.039
Mild-to-moderate DFUs, n (%)	153 (43.3)	139 (39.0)	125 (35.8)	
severe DFUs, n (%)	200 (56.7)	217 (61.0)	225 (64.2)	
HB (g/L)	117 (102,128)	113 (101,127)	115 (104,129)	0.232
ALB (g/L)	33.4 (30.3,37.1)	33.3 (30.1,36.9)	35.1 (31.7,38.3)	< 0.001
HbA1c (%[mmol/mol])	8.0 (7.1,9.6)	8.5 (7.4,10.1)	9.2 (8.1,11.0)	< 0.001
Duration of diabetes ≥ 10 years, n (%)	240 (68)	245 (68.8)	249 (71.1)	0.643
TC (mmol/L)	3.8 (3.1,4.9)	4.0 (3.3,4.8)	4.2 (3.4,5.1)	0.002
LDL-C (mmol/L)	2.0 (1.5,2.6)	2.2 (1.8,2.8)	2.4 (1.8,3.2)	< 0.001
HDL-C (mmol/L)	0.9 (0.8,1.0)	0.9 (0.7,1.1)	0.9 (0.7,1.1)	0.157
eGFR	96.9 (70.9,126.7)	89.5 (58.0,120.6)	82.5 (52.4,114.2)	< 0.001

*BMI* body mass index, *PAD* peripheral artery disease, *HB* hemoglobin, *ALB* albumin, *TC* total cholesterol, *LDL-C* low-density lipoprotein cholesterol, *HDL-C* high-density lipoprotein cholesterol, *eGFR* estimated glomerular filtration rate

### Logistic regression analysis between TyG index and severity of DFUs

The multivariable logistic analysis was performed to explore the relationship between TyG tertile and severity of DFUs (Table 2). Comparing with tertile 1 as reference, the tertile 3 was related to the presence of high score DFUs in the unadjusted model (OR = 1.377 [95% CI: 1.017–1.865], *P* = 0.039). Furthermore, the significant difference of this association still existed in the fully adjusted model (OR = 1.506 [95% CI: 1.079–2.103], *P* = 0.016).

**Table 2:** The relationship between TyG index and the severity of DFUs

Outcomes	Model 1		Model 2		Model 3		Model 4	
	OR (95% CI)	P	OR (95% CI)	P	OR (95% CI)	P	OR (95% CI)	P
T1	Ref		Ref		Ref		Ref	
T2	1.194(0.885,1.611)	0.245	1.201(0.890,1.621)	0.231	1.232(0.911,1.667)	0.175	1.261(0.926,1.716)	0.141
T3	1.377(1.017,1.865)	0.039	1.372(1.012,1.860)	0.041	1.396(1.027,1.898)	0.033	1.506(1.079,2.103)	0.016

Model 1 was unadjusted

Model 2 was adjusted for age, gender

Model 3 was adjusted for age, gender, BMI, smoking, alcohol, PAD, duration of diabetes ≥ 10 years

Model 4 was adjusted for age, gender, BMI, smoking, alcohol, PAD, duration of diabetes ≥ 10 years, HB, ALB, HbA1c, eGFR, serum lipid levels

### Subgroup analysis on association between TyG index and severity of DFUs

To further investigate the impact of other risk factors on the association of TyG index with severity of DFUs, subgroup analyses were carried on according to the following stratification variables: age, sex, BMI, smoking, drinking and PAD. The results of subgroup analyses and interactions were summarized in Table 3. No additional interaction was observed between TyG index and severity of DFUs. More pronounced effects were found among men, patients with age ≥ 65 years, duration of diabetes more than 10 years, or without PAD.

**Table 3:** Subgroup analysis of the association between the TyG and DFUs

Variables	No. of participants	adjusted OR(95% CI)	P	P for interaction
Age				0.197
< 65				
T1	133	Ref	Ref	
T2	137	0.887(0.531,1.481)	0.646	
T3	153	1.059(0.637,1.762)	0.824	
≥ 65				
T1	220	Ref	Ref	
T2	219	1.532(1.039,2.258)	0.031	
T3	197	1.821(1.204,2.755)	0.005	
Gender				0.335
Male				
T1	231	Ref	Ref	
T2	238	1.355(0.929,1.977)	0.115	
T3	211	1.793(1.192,2.695)	0.005	
Female				
T1	122	Ref	Ref	
T2	118	0.965(0.559,1.663)	0.897	
T3	139	0.959(0.563,1.635)	0.878	
BMI				0.491
< 24				
T1	202	Ref	Ref	
T2	204	1.469(0.974,2.216)	0.067	
T3	202	1.533(1.004,2.340)	0.048	
≥ 24				
T1	151	Ref	Ref	
T2	152	1.025(0.644,1.633)	0.916	
T3	148	1.457(0.899,2.362)	0.127	
Smoking				0.72
Yes				
T1	95	Ref	Ref	
T2	118	1.205(0.675,2.153)	0.528	
T3	90	1.757(0.928,3.327)	0.084	
No				
T1	258	Ref	Ref	
T2	238	1.305(0.904,1.884)	0.155	
T3	260	1.401(0.968,2.028)	0.074	
Alchohol				0.799
Yes				
T1	94	Ref	Ref	
T2	116	1.100(0.619,1.953)	0.746	
T3	81	1.662(0.858,3.220)	0.132	
No				
T1	259	Ref	Ref	
T2	240	1.314(0.911,1.894)	0.144	
T3	269	1.440(1.000,2.075)	0.05	
PAD				0.367
Yes				
T1	263	Ref	Ref	
T2	266	1.1107(0.777,1.578)	0.573	
T3	227	1.299(0.889,1.898)	0.176	
No				
T1	90	Ref	Ref	
T2	90	1.749(0.936,3.266)	0.08	
T3	123	2.111(1.152,3.869)	0.016	
Duration of diabetes				0.773
≥ 10 years				
T1	240	Ref	Ref	
T2	245	1.339(0.925,1.939)	0.122	
T3	249	1.515(1.035,2.217)	0.033	
< 10 years				
T1	113	Ref	Ref	
T2	111	1.080(0.614,1.900)	0.79	
T3	101	1.445(0.797,2.622)	0.226	

## Discussion

In this study, we retrospectively investigated the relationship between the TyG index and severity of DFUs in Southeast of China and revealed that the TyG index was significantly associated with the Wagner classification, independent of conventional diabetic foot risk factors, including age, gender, BMI, smoking, alcohol, PAD, duration of diabetes ≥ 10 years, HB, ALB, HbA1c, eGFR, TC, LDL-C, HDL-C. Similar results were found in the subgroup analysis, further emphasizing the robustness of these associations. To our best knowledge, the present study is the first study to explore the relationship between the TyG index and the severity of DFUs.

TyG index is a simple surrogate for IR, which has been implicated as a factor in cardiovascular disease [20–23]. Accumulating retrospective studies have indicated that high TyG index levels seemed to be associated with an increased risk of major adverse cardiovascular events (MACEs) [24–27]. Moreover, recent data showed that higher TyG index reflected a more severe IR and was associated with poor outcomes due to all-cause and cardiovascular disease in a non-linear manner [28–30], suggesting that insulin resistance plays a promoting role in the pathogenesis of cardiovascular and metabolic diseases [31]. Furthermore, Zhao, S., et al. [32] reported that an elevated TyG index was significantly related to a higher risk of both marco- and microvascular damage. This conclusion is in favor of the clinical importance of the TyG index in assessing peripheral vascular injury.

In the last years, the prevalence of PAD has been reported approximately in 65–70% of patients with ischaemic DFUs being a current issue for professionals involved in the management of diabetic foot disease [33]. Previous studies have reported the relationship between TyG index and PAD, which is one of pathogenesis of diabetic foot. A study of a small retrospective study of 71 participants showed that TyG index was an independent biomarker of PAD [34]. Moreover Gao, J.W., et al. demonstrated that Higher TyG index is independently associated with an increased risk of incident PAD based on a large sample size of 12,320 PAD patients [35]. However, they draw a conclusion in their study based on a non-diabetic patient population. While in our study, we indeed found that higher TyG index was correlated to higher Wagner classification, which referred to the presence of more severe diabetic foot ulcers. Interestingly, no significant difference was observed in PAD in the logistic regression models. This can be attributed to failure to adjust the mild PAD that may not influence the lower extremity blood flow because we didn’t stratify the severity of PAD.

Subgroup analysis and exploration of interactions is critical for clinical research, to better understand the actual relationships between independent variables and dependent variables. In this study, these factors, including age, sex, BMI, smoking, drinking, PAD and duration of diabetes, were taken as stratified variables, and more pronounced effects were observed in participants with age ≥ 65 years, duration of diabetes more than 10 years, or in males, or without PAD. The results demonstrated that patients who were elder and had longer duration of diabetes were more predisposed to develop more severe diabetic foot ulcers. A prospective cohort study conducted by Chu, Y.J., et al. reported that those who were more than 70 years were considered as an independent predictor of re-amputation, mortality and functional decline in daily life [36]. The results of our study found the severity of PAD did not interact with the association between TyG and severity of DFUs significantly, although more pronounced effect of TyG on DFUs can be observed in patients without PAD. We hypothesize that there were more confounding factors in patients with PAD, such as more complications, interventions, and utilization of drugs related to hyperlipidemia, hypertension, and antiplatelet, which would attenuate the relationship between TyG and severity of DFUs in PAD subgroup. The current studies suggested that diabetic foot and amputation prevention strategies should focus on men, working freelancers and those with long-term diabetes [37–39]. Increasing knowledge with regard to diabetic foot would help avoid further complications and reduce the occurrence of DFUs. On the whole, based on subgroup analysis, the TyG index appeared to be more sensitive for predicting risk of diabetic foot ulcer in elder male individuals, suggesting it may be promising for screening risk of future diabetic foot ulcers, Therefore, systematic and individualized education should be provided to improve the self-care practice related to diabetic foot prevention in patients with diabetes to avoid further poor outcome.

The strength of this study is that we use an available and inexpensive biomarker to explore the relationship between TyG index and the severity of diabetic foot ulcers based on large populations. Nonetheless, this study also has some limitations. First, this study is a retrospective cross-sectional observational study. Given that TyG index levels may vary over time, the dynamic TyG index should be measured in the following clinical work. What’s more, due to the cross-sectional study, we could not determine the causal relationship between TyG level changes and PAD occurrence. And we provide a reasonable hypothesis that most PAD patients administered hypolipidemic or antiplatelet agents to manage of lipid levels to prevent further advancement of arteriosclerosis. These two factors may lead to the higher lever in TyG Tertile1 and no interaction between PAD subgroup. Second, HIEC, the gold standard method for measuring IR, was not used to compare with the TyG index. However, a large number of studies mentioned above have demonstrated the relationship between the TyG index and IR, as measured by the HIEC. Third, other confounding factors such as exercise habit, nutritional status and medication were not included in the model. Fourth, limitation of sample size may account for no significant difference in the interaction between TyG and other variables.

## Conclusion

In conclusion, we found higher triglyceride glucose index is independently associated with severity of diabetic foot ulcers in individuals with diabetes even after adjusting for conventional risk factors. In this instance, TyG index may serve as a helpful tool in the assessment of DFUs. Furthermore, the potential mechanism of the relationship between the TyG index and the severity of DFUs requires further prospective large-scale exploration.

## Data Availability

The datasets analyzed during the current study are available from the corresponding author upon reasonable request, provided appropriate credit is attributed to the original authors and the data source.
